# Crosstalk between Bone and Muscles during Physical Activity

**DOI:** 10.3390/cells12162088

**Published:** 2023-08-18

**Authors:** Luca Dalle Carbonare, Arianna Minoia, Sharazed Zouari, Francesca Cristiana Piritore, Anna Vareschi, Maria Grazia Romanelli, Maria Teresa Valenti

**Affiliations:** 1 Department of Engineering for Innovative Medicine, University of Verona, 37100 Verona, Italy; luca.dallecarbonare@univr.it (L.D.C.); arianna.minoia@univr.it (A.M.); sharazed.zouari@univr.it (S.Z.); anna.vareschi@univr.it (A.V.); 2 Department of Neurosciences, Biomedicine and Movement Sciences, University of Verona, 37100 Verona, Italy; francescacristiana.piritore@univr.it (F.C.P.); mariagrazia.romanelli@univr.it (M.G.R.)

**Keywords:** physical activity, bone, muscle, progenitor cells, extracellular vesicles

## Abstract

Bone–muscle crosstalk is enabled thanks to the integration of different molecular signals, and it is essential for maintaining the homeostasis of skeletal and muscle tissue. Both the skeletal system and the muscular system perform endocrine activity by producing osteokines and myokines, respectively. These cytokines play a pivotal role in facilitating bone–muscle crosstalk. Moreover, recent studies have highlighted the role of non-coding RNAs in promoting crosstalk between bone and muscle in physiological or pathological conditions. Therefore, positive stimuli or pathologies that target one of the two systems can affect the other system as well, emphasizing the reciprocal influence of bone and muscle. Lifestyle and in particular physical activity influence both the bone and the muscular apparatus by acting on the single system but also by enhancing its crosstalk. Several studies have in fact demonstrated the modulation of circulating molecular factors during physical activity. These molecules are often produced by bone or muscle and are capable of activating signaling pathways involved in bone–muscle crosstalk but also of modulating the response of other cell types. Therefore, in this review we will discuss the effects of physical activity on bone and muscle cells, with particular reference to the biomolecular mechanisms that regulate their cellular interactions.

## 1. Physical Activity and Wellness

The fact that bone is an endocrine organ responsible for regulating energy metabolism has confirmed the understanding that bone-derived hormones play a crucial role in regulating muscle functions and facilitating adaptation to exercise [1]. Exercise has been found to trigger the secretion of proteins from various tissues, including skeletal muscle and bone. These proteins may play a role in the positive effects of physical activity and offer potential opportunities for therapeutic interventions to harness the benefits of exercise [2,3].

It is well known that physical activity plays an important role in both improving mental health and well-being and in the prevention and treatment of degenerative diseases [4,5].

Muscle and bone can be affected by pathologies such as sarcopenia and osteoporosis, respectively, and the increase in life expectancy and sedentarism will increasingly contribute to the development of these diseases. Sarcopenia is due to disorders that cause a high and accelerated reduction of muscle mass and function, while osteoporosis is a skeletal pathology represented by reduced bone mass and poor quality of bone. In recent years, a correlation between sarcopenia and osteoporosis has been suggested [6]. Thus, osteosarcopenia can be defined as a syndrome whose pathogenesis involves genetic polymorphisms, endocrine alterations, reduction in the mechanical load or impaired crosstalk between muscles, bones and fat cells [6].

Exercise can improve the number and quality of stem cells that give rise to muscle or bone. Stem cells play an important role in ensuring a better quality of life for both young and elderly individuals [7]. Numerous studies advocate exercise as a potential therapeutic approach to enhance muscle and bone health in individuals with diseases, aiming to mitigate the adverse effects of these conditions [8,9,10,11,12,13,14].

The role of cytokines as well as of non-coding RNAs in promoting crosstalk between bone and muscle has been demonstrated not only in physiological conditions, referred to as the body’s baseline state of health, or following bone and muscle pathologies, but also in response to stimuli deriving from lifestyle such as physical activity which requires energy expenditure. On the contrary, a sedentary lifestyle contributes significantly to metabolic alterations and, therefore, to an increase in pathologies such as cardiovascular diseases or those linked to skeletal or muscle degenerative processes.

Above all, considering the importance of physical activity for preventing non-communicable diseases, such as obesity, hypertension or high cholesterol levels, it is clear that promoting physical activity is a winning strategy to reduce the burden on healthcare systems and promote overall well-being.

## 2. Progenitor Cells

The remarkable capacity of tissues to uphold their function and undergo self-restoration following injury can be attributed to the existence of resident adult stem cells. These cells remain in a dormant state within all tissues and, when prompted by suitable circumstances, possess the ability to activate themselves, replenishing depleted cells [15].

In the mammalian embryo, the myogenesis involves the expression of *Pax3* and *Pax7*, which are two paired-homeobox transcription factors crucial for myogenic lineage specification in muscle progenitor cells [16]. These progenitor cells shape a satellite position close to the myofiber and are called muscle satellite cells (MuSCs). Thus, satellite cells are resident muscle stem cells with a great regenerative capacity. These cells remain in a dormant state and re-enter the cell cycle to regenerate muscle tissue and refill the stem cell pool [17,18]. Muscle tissue interacts with bone tissue, which provides it with attachment sites for muscles, tendons, and ligaments. Bone tissue also has great regenerative capacity; it can, in fact, repair fractures by restoring damaged bone to a pre-injury functional state [19]. This ability of the bone remodeling process is due to mesenchymal stem cells (MSCs), which are multipotent cells able to differentiate into chondrocytes, osteoblasts, or adipocytes. The differentiation process that leads these cells to differentiate into different cell types depends on different cellular pathways such as Wnt, TGF-β, BMP, and FGF [20].

The natural aging process is characterized by a progressive loss of homeostasis, which results in a variety of physiological alterations in cell and tissue function [21]. Importantly, regenerative capacity decreases with aging [5]. Reduced regenerative capacity as a result of aging is due to a decrease in the number and function of stem cells [7] while aged satellite cells are activated more slowly than young satellite cells [22].

In this context, physical activity positively influences the activation of stem cells. Exercise plays a significant role in influencing the destiny of mesenchymal stem cells (MSCs). Signals generated during exercise trigger a shift in MSC differentiation, leading to a preference for osteogenesis (bone formation) over adipogenesis (fat formation). Notably, the activation of the Wnt signaling pathway during exercise prompts the secretion of pro-osteogenic proteins, including the Runt-related transcription factor 2 (RUNX2). This molecular mechanism reinforces the promotion of bone-building processes in response to physical activity) [23]. The study conducted by Maredziak et al. confirmed that exercise increased the total number of MSCs in the bone marrow cavity in mouse models [24]. It has also been observed that osteoblast precursors have a higher osteogenic potential in active animals than in sedentary animals [24]. In particular, it has been observed that exercise activates the ERK1/2 pathway, involved in various cellular processes, including cell differentiation, and reduces the expression of PPAR-γ, a trancription factor that plays a significant role in adipogenesis, highlighting how exercise modulates the differentiation of MSCs toward osteogenesis compared with adipogenesis [24].

Hsu et al. demonstrated that the number of satellite cells increases following training [25]. It has also been demonstrated that a single session of resistance exercise (RE) together with high intensity training (HIIT) increases the number of satellite cells in type I fibers by 78% t in sedentary, overweight subjects; this may help reduce the loss of muscle mass that occurs with aging [26].

Increased expression of satellite cell transcription factors such as Pax7, NCAM, FA1, Myf5, MyoD and myogenin was observed in 10 healthy young men who underwent 12 weeks of resistance training. The results of this study show that exercise increases the number of satellite cells but also their activation for tissue regeneration and repair due to the increase in NCAM and myogenin [27].

Exercise can restore certain patterns of genes that may be important for satellite cell function. It has been observed that in muscle stem cells after exercise, cyclin D1 deficiency led to the transcription of pro-aging genes such as *TGF-β* and increased the expression of cyclin D1, thus improving satellite cell activity [28].

Thus, by evaluating the effects of a half-marathon on amateur participants, we recently demonstrated that physical activity improves both myogenic and osteogenic stem cells population [29,30].

## 3. Bone and Muscle Cytokines

Bone and muscle interact either mechanically or chemically [31] and bone and muscle homeostasis are strongly connected [32]. In particular, bone and muscle crosstalk through the endocrine system secreting myokines such as irisin, IL-6 and myostatin and osteokines such as sclerostin.

**Figure 1:**
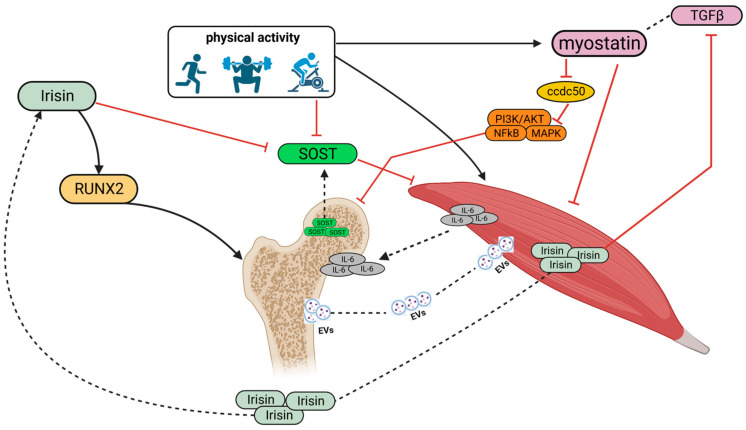
Physical activity stimulates the production and secretion of myokines such as irisin, IL-6 and myostatin which affect bone and muscle homeostasis. In particular, myostatin and irisin have an opposite action in bone–muscle metabolism: physical activity primarily stimulates the release of myostatin, which is part of the TGFβ-superfamily, induces osteoclast genesis downregulating *Ccdc50* and protein degradation in skeletal muscle; subsequently, physical activity promotes the release of irisin which stimulates osteogenic and skeletal differentiation, inhibiting TGFβ-signaling and the SOST gene; the latter is also inhibited by the effect of physical activity. The production of IL-6 induced by physical activity promotes the differentiation of satellite cells and osteoblast differentiation. Extracellular vesicles mediated the bone and muscle crosstalk as well. Created with BioRender.com accessed on 22 June 2023.

Exercise induces myocytes to secrete myokines, which control many metabolic processes in various tissues including bone. Irisin, interleukin 6 (IL-6), and myostatin are among the major myokines produced [33,34]. Skeletal muscle secretes irisin during exercise, which can increase osteoblast differentiation in vivo (Figure 1). In the study conducted by Colaianni et al., muscle cells were obtained from mice undergoing a 3-week training program; These muscle cells were cultured for 14 days to obtain a conditioned medium, which was subsequently used to culture bone marrow stromal cells. Thus, bone marrow stromal cells cultured in this conditioned medium exhibited a strong tendency toward osteogenic differentiation [35]. Irisin plays a crucial role in osteogenic differentiation and bone health; therefore, some research groups have investigated how irisin acts at the molecular level. The study conducted by Sun et al. found that irisin administration enhances RUNX2 expression through inhibition of TGF-β/Smad signaling, promoting osteogenesis and regulating osteoblast differentiation. This inhibition depends on irisin binding to the TGF-β receptor II (TβRII), competing with TGF-β and preventing activation of the signaling pathway [36]. A further study showed that irisin can promote upregulation of pro-osteoblastic genes and reduce levels of osteoblastic inhibitors such as osteokine sclerostin, mainly produced by osteocytes, thus improving bone formation and density [37]. In particular, sclerostin inhibits bone formation by the canonical Wnt/β-catenin signaling pathway [32]. Importantly, the Wnt/β-catenin signaling pathway also plays an important role in skeletal muscle regeneration and elevated levels of sclerostin in serum have been associated with low skeletal muscle mass [38]. These findings are very important for understanding how physical activity promotes the crosstalk between muscles and bones. Thus, irisin, produced by the muscle following exercise, in addition to promoting bone formation, has an important role in muscle regeneration.

Myostatin and irisin play contrasting roles in regulating muscle–bone homeostasis, exerting their effects through the IGF-1/Akt and mTOR pathways. These pathways are crucial in governing cellular proliferation and survival or energy metabolism [39]. While irisin is promyogenic, myostatin reduces protein synthesis and induces protein degradation in skeletal muscles. Myostatin belongs to the transforming growth factor-β (TGF β) superfamily and, in addition to negatively regulating muscle mass, also decreases bone mass through increased osteoclastogenesis [40]. Indeed, studies have shown that myostatin induces osteoclastogenesis, leading to increased bone resorption. This effect is achieved by downregulating the gene expression of *Ccdc50*, which in turn affects the PI3K/Akt, MAPK, and NF-κB pathways [41]. Śliwicka et al. reported that a marathon race performed by 10 amateur runners induced an increase in myostatin and irisin and sclerostin levels [39]. In particular, the authors observed that irisin levels were increased and reached their highest levels 72 h after the marathon while myostatin levels were significantly increased 24 h after the marathon and sclerostin levels increased only 72 h after the marathon [39]. The authors suggest that the increase in myostatin, which has a negative effect on muscle, is due to its involvement in inflammation.

Working skeletal muscle is able to produce interleukin-6 (IL-6) and the production levels are associated with the duration and intensity of the exercise [42]. Moreover, it has been observed that exercise induces the secretion of IL-6, which plays a crucial role in promoting satellite cell differentiation. This observation has significant importance as the absence of IL-6 leads to a decline in the differentiation capacity of satellite cells due to a decrease in STAT3 activation in these cells [43]. IL-6 is mainly involved in inflammation processes but also participates in bone remodeling through an increase in osteoclast formation and osteoblast differentiation [44].

Moreover, in addition to cytokines secretion, extracellular vesicle-mediated crosstalk, as we will discuss later, significantly contributes to the interaction between bones and muscles during physical activity.

## 4. Extracellular Vesicles Modulation during Physical Activity

It has been well reported that exercise stimulates the release of many molecules packaged in extracellular vesicles (EVs), supporting the concept that these structures play a crucial role in adaptation to exercise [45]. EVs are composed of lipid bilayer membranes and are actively secreted by a wide variety of cells. These structures facilitate the transfer of lipids, proteins, and nucleic acids, and they have the potential to affect various pathophysiological processes in both the cells that release them (parent cells) and the cells that uptake them (recipient cells) [46].

EVs can be categorized into different subtypes based on their size. These subtypes include exosomes (ranging from 20 to 150 nm in diameter), microvesicles (ranging from 100 to 1000 nm in diameter), and apoptotic bodies (ranging from 500 to 5000 nm in diameter). Among these, exosomes are the most extensively studied and best-characterized subtype [47]. EVs carry a significant cargo of miRNA, which are single-stranded, non-protein-coding RNAs approximately 22–26 nucleotides in length. These miRNAs act as regulators at the transcriptional or post-transcriptional level by binding to the 3′-untranslated regions of mRNA transcripts [48]. MiRNAs can modulate crucial steps in satellite cell renewal, skeletal muscle plasticity as well as in regeneration processes. They also are involved in gene expression changes associated with sarcopenia and in facilitating skeletal muscle adaptation to exercise in the elderly population [49].

Recent studies have shed light on the significant functions of exosomes in bone and muscle cells, emphasizing their role in facilitating communication between these two tissues [50]. While mechanical loading via muscle contraction was once considered the primary mechanism by which muscle influences bone mass [51], emerging research indicates that extracellular vesicles, carrying microRNAs derived from skeletal muscle and bone cells, can also be transported and contribute to the regulation of bone–muscle crosstalk [52]. MiR-27a-3p-containing exosomes of C2C12 myoblasts have also been reported to promote osteogenic differentiation [53].

During moderate exercise, the contraction of muscles not only improves bone quality but also extends lifespan [53]. A significant number of EVs are present in the bloodstream, with muscle-derived EVs accounting for approximately 1–5% of the total circulating EVs. During exercise, muscle-specific microRNAs enclosed within these EVs are released into the bloodstream, and around 60 to 65% of the muscle-derived EVs are CD81 positive [54].

In the elderly, the intensity and frequency of exercise are often reduced, leading to changes in the cargo of senescent cell-derived EVs. In addition, it has been reported that these alterations in EV content contribute to an age-related decline in somatic function [17]. Skeletal muscle undergoes increased oxidative stress as it ages, leading to a rise in the expression of the senescence-associated microRNA, miR-34a. This heightened expression of miR-34a is observed both in skeletal muscle itself and in muscle-derived EVs [52]

MiR-34a expression is also increased in exosomes from C2C12 cell line myoblasts subjected to oxidative stress [55]. In particular, the study highlighted that EVs isolated from the medium of H2O2-stressed C2C12 cells showed a significant increase in miR-34a levels. Remarkably, these EVs triggered senescence in bone marrow-derived mesenchymal stem cells (BMSCs) by downregulating the expression of SIRT1.

In the aging muscle, levels of miR-34a are elevated and can contribute to impaired muscle metabolism, while reduced SIRT1 activity is associated with decreased muscle performance [56]. EVs originating from C2C12 myoblasts or myotubes exhibit the capability to enhance osteogenic differentiation in MC3T3-E1 cells by activating the β-catenin cellular pathway through the expression of miR-27a-3p [57]. Notably, miR-27a has also been shown to promote myoblast differentiation [58]. Furthermore, there is evidence suggesting that muscular EVs exert an influence on osteoclast formation. As a result, these findings underline the significant role played by miR-27a contained within muscle-derived EVs in facilitating communication between bone and muscle tissues [59].

Hence, exercise effectively modulates cellular pathways not only within skeletal muscles but also in other organs, yielding beneficial systemic metabolic effects [60]. In the context of an acute exercise session, proteome analyses performed on EVs extracted from the plasma of healthy male subjects revealed an increase in EV-derived proteins and small EVs. Notably, the levels of these molecules returned to their baseline after a span of 4 h [45]. Furthermore, insights obtained by mouse model studies have convincingly demonstrated that supplementation with exosomal miRNAs derived from the muscles of trained mice enhances glucose tolerance in sedentary mice [61].

In summary, these investigations strongly suggest that exosomes triggered by exercise (referred to as “exersomes” [62]), together with the bioactive cargo they transport, hold great promise as potential targets for therapeutic interventions aimed at counteracting metabolic disorders.

## 5. Interconnected Bone and Muscle Molecular Pathways

**Figure 2:**
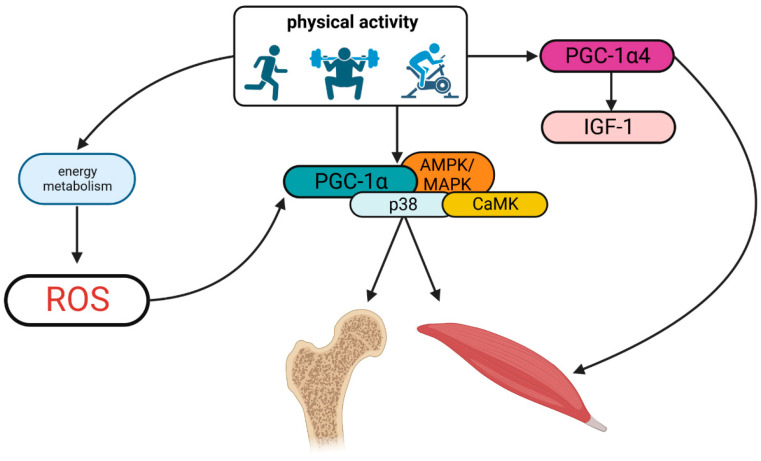
Physical exercise effects on musculoskeletal cells. Created with BioRender.com accessed on 22 June 2023.

Muscles and the gravitational field exert forces on the bone during locomotion and exercise [63]. Physical activity is a key way to enhance bone strength during the developing years because it influences the density and structural qualities of bone [64]. Additionally, exercise can increase skeletal muscle mass, a key factor in bone strength. Because of mechanical loading resulting from gravitational load and muscular contractions, physical activity through skeletal muscle have been shown to improve bone characteristics [65]. The Frost’s Mechanostat Theory describes this mechanism: it contends that mechanical loading above a predefined threshold range, which causes bone strains, promotes bone to become stronger by altering its mass, density, and structure [66]. Additionally, muscle may control bone modelling via endocrine and paracrine signaling mechanisms [67]. Physical exercise, in general, leads to the activation of ROS (reactive oxygen species) which induce the activation of multiple intracellular signal pathways that are responsible for the benefit of exercise in muscles [68]. Physical exercise not only has a positive effect on musculoskeletal cells but also elicits positive responses in various other tissues, including adipose tissue, endothelium, the central nervous system, and endocrine organs [69]. It has been common to think that ROS produced during physical activity had a negative effect on health [70]. In the past decade, a new perspective has emerged, recognizing reactive oxygen species (ROS) as important mediators of signal transduction pathways (Figure 2).

Several factors may trigger skeletal muscle to produce more ROS after exercise [68]. Exercise increases the need for ATP synthesis, which in turn activates energy metabolism due to the presence of ROS. This is due to high rates of ATP hydrolysis, purine metabolism, and cytoplasmic glucose metabolism as well as NADH regeneration [71]. Fast-twitch muscle fibers, which contain high levels of glycogen and use glycolysis as their primary source of ATP, are the main sources of lactate formation and release [72]. Skeletal muscle receives a variety of physiological stimuli resulting from different types of exercise, though all types are regulated by factors such as the volume and intensity of contractile stimuli, together with the exercise-induced protein’s half-life. PGC-1 (Peroxisome proliferator-activated receptor-gamma coactivator (PGC)-1alpha) has emerged as a pivotal mechanistic connection to a myriad of beneficial effects of exercise on muscle including mitochondrial biogenesis, angiogenesis, the utilization of fatty acids, the change in fiber type and antioxidant defense [73,74]. Other cellular signaling pathways, like p38 mitogen-activated protein kinase (MAPK)/5-adenosine monophosphate-activated protein kinase (AMPK) and Ca^2+^/calmodulin-dependent protein kinase (CaMK), similarly cause a rise in PGC-1 mRNA levels following muscular contraction [75,76]. Recent research has shown that antioxidant therapy inhibits lactate-induced PGC-1 in C2C12 myoblasts, demonstrating that ROS may synergize with lactate metabolism to promote PGC-1 synthesis. Moreover, these findings suggest that lactate can stimulate mitochondrial biogenesis within skeletal muscle [68,77].

Exercise is typically divided into two categories: resistance training and endurance training. Both categories can induce the expression of PGC-1α4, a variation truncated isoform of PGC-1α [74]. This isoform has been found to enhance the expression of IGF-1 and to be associated with hypertrophic muscle response [78]. The loss of muscle mass and strength that occurs with age and muscular dystrophies has an effect on both quality of life and longevity. PGC-1α4 could control both of these systems in a highly coordinated manner. Additionally, improving resistance training with a focus on boosting muscle strength may be aided by monitoring *PGC-1α4* gene expression [78]. Thus, the current observations locate PGC-1α as a crucial player in regulating the endurance (oxidative) as well as the resistance (myogenic) adaptations to exercise. Additionally, PGC-1α is crucial for maintaining bone growth and osteogenic differentiation. Numerous studies have shown that PGC-1α-SOD2 signaling is essential for the regulation and production of mitochondria [79]. Studies have examined the role of the SIRT3 protein deacetylase, produced in osteoblasts, in controlling PGC-1α, which was linked to a protein–protein interaction. Reduced PGC-1α protein stability resulted in increased ROS levels, changed mitochondrial activity and aberrant osteogenic differentiation [80]. Moreover, SIRT3 contributes significantly to muscle maintenance by controlling insulin resistance and ROS generation in the mitochondria [81]. Additionally, it has been shown that regular exercise causes an adaptation and a decline in ROS. Because ROS are produced more frequently when TGF-beta-1 (TGF-β1) is present in low concentrations, this would result in increased fitness. Consistent exercise starts a process of adaptation that makes antioxidant enzymes work harder to prevent TGF-β1 activation [82]. TGF-β1 and bone morphogenic protein (BMP) signaling are also crucial for chondrogenic and osteogenic differentiation. Through the TGF-β1/BMP pathways, chondroblasts are stimulated to proliferate and produce extracellular matrix elements (ECM) unique to cartilage, such as aggrecan, COL2A1, and glycosaminoglycans (GAG) [83]. On the other hand, reducing oxidative stress induced by physical activity in bone cells could fight the bone loss with estrogen deficiency and aging, and promote osteogenic differentiation [84].

Several miRNAs have been identified as key regulators of osteogenic or myogenic differentiation (Table 1).

For example, the expression of miR-21 partially restores MSC osteogenesis that has been hampered by TNF. Spry1 expression is downregulated by inhibition of the TNF-signaling pathway, which raises the possibility that miR-21 promotes bone formation by targeting Spry1 in MSCs [85]. miR-21-5 is also involved in the proliferation and differentiation processes of skeletal muscle satellite cells by targeting KLF3 [86].

A modulation of several miRNAs has been demonstrated in patients who participated in a half-marathon. Recently, we showed that the expression of miRNAs involved in osteogenic differentiation such as miR-21-5p, miR129-5p and miR-378-5p was increased following a half-marathon. In addition to miR-21-5p, miR129-5p and miR-378-5p are also involved in myogenesis [49,87,88].

MiR143 modulation was additionally observed in the participants in the half-marathon. This microRNA serves as a regulator of insulin-like growth factor-binding protein 5 (IGFBP-5). Disruptions in the expression of miR-143 and its corresponding target gene have been noted in primary myoblasts, thus impacting the process of muscle regeneration [29]. Moreover, the TGF-β/miR-143/145 axis in skeletal muscle cells demonstrated that miR-143 is upregulated during physical activity, in response to increased TGF-β signaling induced by miR143, which reduces insulin signaling and impairs muscle performance [89]. Interestingly, it has been demonstrated that miR-143, by targeting HDAC7, promotes the differentiation of osteoblast [90].

Furthermore, it has been demonstrated that a modulation in circulating miRNAs in participants in a half-marathon leads to an increase in the expression of MYOD, a transcription factor for myogenic differentiation, as well as of RUNX2, the transcription factor for osteogenic differentiation [29,30].

## 6. In Vitro and In Vivo Model to Evaluate the Effects of Physical Activity on the Bone–Muscle Crosstalk

Numerous studies have utilized in vivo models to examine the impact of physical activity on the interaction between bones and muscles and several studies have evaluated individuals engaging in various levels of physical activity to assess the effects of exercise on the bone–muscle crosstalk [91]. Most of the time, the impacts of physical activity are evaluated across a range of age groups or/and activity intensity to evaluate how they affect bone metabolism and correlate with the growth of muscle mass [65].

It has been suggested that maintaining muscle strength even beyond childhood is crucial for improving bone metabolism in adults [92]. In this context, a study explored the potential links between distinct patterns of objectively measured physical activity and bone parameters during late adolescence. Furthermore, this investigation deepened the mediatory impacts of lean soft tissue, which serves as a representative measure of muscle mass, on these relationships. The study used specialized sensors that can detect force through the acceleration generated. The findings suggest that interventions involving physical activity during the growth phase improve muscle mass, particularly in women, contributing to improved bone health in the proximal femur [65].

Analyses carried out on affected tissues can also be of help in fully understanding the effects of physical activity. By analyzing mesenchymal circulating progenitor cells, we recently reported that high-intensity physical activity influences the osteogenic metabolism leading to a greater expression of *RUNX2*, the master gene of osteogenic differentiation [93].

Besides examining the direct effects of physical activity on human subjects, researchers also utilize model organisms to investigate the impacts of exercise. As far as the use of mice is concerned, it is often linked to studies in which the benefit of the activity is analyzed in models with deficits related to muscle and bone metabolism [94]. In *mouse* models affected by sarcopenia, the beneficial role of sirtuin 1 in muscle cell development and maturation and subsequent force production has been highlighted [95].

In a mouse model with induced muscle injury, researchers observed how physical activity affects the production of osteogenesis and chondrogenesis factors [96]. In a previous study by Fry CS et al., satellite cells have been shown to be necessary for the maintenance of physical function and for the increase in muscle fiber size in response to physical activity throughout life. Satellite cells are not required for immediate adaptations to a growth stimulus in adult mice as well as for the preservation of muscle mass in sedentary aging mice [97]. The authors shed new light on the specific contribution of satellite cells to skeletal muscle hypertrophy resulting from long-term physical activity. They suggest that preserving the composition of satellite cells during aging could potentially enhance muscle growth and maintain physical performance in older individuals participating in regular physical activity [98].

In addition, the *zebrafish* model was employed to investigate the relationship between bone–muscle interactions and lipid metabolism [99]. The ongoing progress in lipidomic profiling offers opportunities for further understanding the effects of lipid-modulating gene expression and other roles in lipid signaling on bone and muscle. Such knowledge is crucial for preserving human health and mitigating the development of diseases [100].

2D-cell cultures are still used as a method for studying how exercise affects osteoblasts, chondroblasts, adipocytes, or muscle cells. But it is worth pointing out that is not always acceptable for 2D-in vitro models to mimic this kind of physical exercise [101].

On the basis of the exercise stimulus (mode, intensity or duration), and if stimulus is executed acutely or in a training routine, the precise mechanisms of exercise-induced reactions are not entirely understood [102]. For an experimental model to be deemed an in vitro exercise-like treatment, it should replicate exercise-induced transcriptional signaling and metabolic changes observed in vivo. Furthermore, it was demonstrated that applying electro pulsing (EPS) to muscle cells serves as a promising in vitro model for simulating the effects of physical activity [103]. This model can be used to better understand how muscle contraction affects skeletal muscle signal transduction [104] and myokine release [104]. EPS can be a helpful in vitro model, but depending on the approach utilized, it can also produce similarities and differences. It can further provide a different greenness of reaction if strength or resistance training is involved. Therefore, it can be helpful to conceive of EPS as a universal model of skeletal muscle contraction that can be employed in either an acute or chronic setting, rather than focusing on any activity [101].

To exploit the correct stimuli induced by the activity on a 2D-cell culture, it is possible to condition 2D cell lines using the serum of active patients. By conditioning mesenchymal cells, physical activity has been shown to promote complex biological interactions, leading to osteogenic and chondrogenic differentiation, as well as the induction of autophagy [105]. Additionally, it was shown that exercise induces the modulation of miRNAs linked to muscle differentiation in both women and men by stimulating progenitor cells with serum collected from subjects following physical activity [29].

It could be interesting to exploit bioreactors such as 3D scaffolds to condition bone and muscle cells with serum from subjects who have conducted physical activity to reverse the effects [106]. Even the isolation of progenitor mesenchymal cells from samples of subjects who have engaged in physical activity may be used in a 3D setting as an environment for the cells’ growth [107]. The development of in vitro exercise systems employing 3-D organoids, tissue-on-a-chip models or bioprinting could result in more sophisticated models for investigating intercellular communication, even with other cell types [108].

Finally, we can conclude that the best evaluation of the effects of physical activity in bone–muscle crosstalk remains evaluating it directly in people who practice, or to perform ex vivo studies exploring the stimuli that have been induced by the activity itself [30,93,105].

## 7. Conclusions

In conclusion, many studies suggest that physical activity induces a complex network of interactions between muscle and bone. Many of these interactions induce an improvement in the proliferative and differentiation processes of the stem cells and therefore an increase in the regenerative potential both in the muscle and in the bone. An increase in the regenerative potential is particularly important to counteract degenerative diseases. Therefore, physical activity and choosing an appropriate exercise program can be considered important tools in the prevention of pathologies related to aging.

## Figures and Tables

**Table 1:** MicroRNAs Functions.

MicroRNAs	Biological Functions	References
• miR-21	Implicated in promoting bone formation targeting Spry1 in MSCs and involved in the proliferation and differentiation processes of muscle satellite cells by targeting KLF3.	[85,86]
• miR-21/miR129/miR-378	Involved in osteogenic differentiation after physical activity and play a role in myogenesis.	[49,87,88]
• miR-143	Upregulated during physical activity with a reduction in IGFBP-5 and is also involved in osteogenic differentiation.	[29,89,90]
